# Delta Storage Pool Deficiency: A Pediatric Case Report and Review of the Literature

**DOI:** 10.7759/cureus.50432

**Published:** 2023-12-13

**Authors:** Jhoan Gonzalez, Juliana Salazar, Alejandra Calderon

**Affiliations:** 1 Hematology and Oncology, Universidad Nacional de Colombia, Bogotá, COL; 2 Medical Physics, Universidad de Los Andes, Bogotá, COL; 3 Pediatric Oncology, Fundación Santa Fe de Bogotá, Bogotá, COL

**Keywords:** secondary hemostasis, primary hemostasis, bleeding child, storage pool deficiency, delta storage pool deficiency

## Abstract

Platelet storage deficiencies are a heterogeneous group of bleeding disorders of variable severity caused by decreased number or content of platelet granules.

We present the case of a 10-year-old patient with no personal history of previous bleeding who was admitted to the emergency department due to menorrhagia and mucocutaneous pallor. Common disorders of primary and secondary hemostasis were ruled out. Subsequently, a study of electron microscopy of platelets was performed, which reported the presence of alpha granules with a decreased number of dense granules.

Currently, the patient receives treatment with tranexamic acid during menstrual periods, supplementation with ferrous sulfate, and oral contraceptives, achieving control of bleeding episodes.

## Introduction

Platelets are the blood cells responsible for primary hemostasis, which aims to generate a primary clot (without fibrin) at the site of vascular injury to stop bleeding [1]. These cells have multiple granules essential for their function, and the most studied ones so far are dense granules and alpha granules, which remain stored until platelet activation triggers the exocytosis of their content [2]. Dense granules mainly contain calcium, adenosine diphosphate (ADP), and serotonin. The released calcium is part of the coagulation cascade, while ADP and serotonin act on specific receptors on the platelet membrane to induce their aggregation [3].

Deficiency of the delta storage pool is a rare group of platelet disorders characterized by a decrease in the amount of dense granules. The diagnosis of this condition is not standardized and requires complex laboratory tests that are not available in all healthcare centers. It should be suspected in patients with suggestive family history and/or clinical presentation of mucocutaneous bleeding, such as hematomas, menorrhagia, gingival bleeding, epistaxis, postoperative bleeding, and postpartum hemorrhage, in which no apparent cause has been found [4].

## Case presentation

A 10-year-old white female patient presents with a two-week history of heavy menstrual bleeding with clots associated with headache, mucocutaneous pallor, and generalized abdominal pain. As for her medical history, one week before the clinical symptoms, she experienced a self-limited episode of epistaxis lasting 4-5 minutes. There were no other relevant bleeding episodes. Two years before the consultation, she underwent a tooth extraction and had a wound on her forehead that required sutures; during these episodes, she did not present with abundant bleeding.

Regarding family history, the patient's sister has a history of menorrhagia, and the patient's mother experienced post-cesarean hemorrhage that was difficult to control. The pictogram of menstrual losses showed a score corresponding to more than 80 ml per cycle. Physical examination revealed tachycardia and mucocutaneous pallor without petechiae, ecchymosis, or hematomas. 

Initial tests showed normocytic, normochromic anemia without thrombocytopenia and normal mean platelet volume. Partial thromboplastin time and prothrombin time were not prolonged, and a transabdominal pelvic ultrasound showed no alterations. Transfusion of packed red blood cells was indicated, and management with tranexamic acid was initiated.

The integrity of both primary and secondary hemostasis was evaluated, as well as connective tissue diseases that could be causing the patient's condition, obtaining normal results (Table 1).

**Table 1:** Studies performed on the patient, with normal results ADP: Adenosine diphosphate; C3: Complement component 3; C4: Complement component 4; IgG: Immunoglobulin G; IgM: Immunoglobulin M; DNA: Deoxyribonucleic acid

Studies	
Hemostasis	Thromboelastography
Primary hemostasis	Platelet count
	Von Willebrand factor antigen
	Von Willebrand factor activity
	Coagulation factor VIII
	Platelet aggregation curves with ADP, collagen, ristocetin, and epinephrine.
	Platelet Function Analyzer 100
Secondary hemostasis	Partial thromboplastin time
	Prothrombin time
	Thrombin time
	Thromboplastin time dilutions
	Coagulation factor XIII
	Reptilase time
	Specific inhibitors of coagulation
fibrinolysis	Plasminogen Activator Inhibitor 1
	Euglobulin lysis time
	Alpha 2 antiplasmin functional
Autoimmune Profile	Total immunoglobulins
	Complement C3 and C4 levels
	Anticardiolipin IgG and IgM
	Beta 2 glycoprotein IgG and IgM
	Lupus anticoagulant
	Antinuclear Antibodies
	Anti-DNA antibodies

During her hospital stay, the patient required transfusions of packed red blood cell units on four occasions, a transfusion of fresh frozen plasma and platelets, and the administration of activated recombinant Factor VII, achieving bleeding control. At discharge, it was indicated to continue with tranexamic acid at a dose of 500 mg orally every 8 hours during menstrual periods, iron supplementation with ferrous sulfate, and oral contraceptives.

During the following 16 months, the patient experienced abundant menstruations; however, they have been controlled with the prescribed medication, with no readmissions to the emergency department.

A study of electron microscopy of platelets was requested, showing no evidence of macro platelets or Döhle bodies in granulocytes, indicating that the patient's condition does not suggest May-Hegglin anomaly, Sebastian syndrome, or Fechtner syndrome. It was reported that there is a presence of alpha granules with a decrease in dense granules. Approximately 10% of the platelets lack dense granules, and 60% have 2 or fewer granules in their cytoplasm. Therefore, the diagnosis of mild deficiency of platelet-dense granules is established. Finally, the patient is referred to clinical genetics for counseling, and they ordered a clinical exome, with results still pending.

## Discussion

During the approach to pediatric patients with bleeding symptoms, a series of tests should be performed to evaluate primary and secondary hemostasis. Starting with basic exams such as a complete blood count to detect the presence of thrombocytopenia and going all the way to more advanced studies such as platelet electron microscopy [5,6]. Among the multiple disorders that can cause bleeding in pediatric age, there are platelet storage pool deficiencies, a group of heterogeneous entities characterized by a decrease or absence of platelet granules, whether alpha (α), dense (δ), or both [7].

Normally, platelets contain between 3 to 8 dense granules, which house adenosine diphosphate, adenosine triphosphate, calcium, and serotonin, among other components. These components are essential for both primary hemostasis, promoting platelet aggregation, and secondary hemostasis, acting as cofactors [3,8]. Dense granules are considered derivatives of lysosomes [8], which explains why the deficiency of dense granules (δ-SPD) can be related to pigmentary abnormalities such as Hermansky-Pudlak Syndrome and Chediak-Higashi Syndrome. This condition can be classified according to its etiology, with hereditary and acquired forms: Under the inherited classification (Type I), subtypes are further specified based on associated characteristics. Includes A, linked with pigmentary abnormalities (Hermansky-Pudlak syndrome and Chediak-Higashi syndrome); B, associated with other hereditary pathologies (TAR syndrome, Wiskott-Aldrich syndrome, and familial leukemia); and C, isolated conditions (autosomal inheritance dominant, empty bag syndrome, and giant dense body disorder). Type I also encompasses D, denoting deficiency of both alpha and delta granules. In the acquired category (Type II), platelet storage pool diseases are correlated with platelet activation, myelodysplasia, and myeloproliferative disorders [9].

The exact prevalence of isolated dense granule deficiency is still unknown due to the lack of specialized diagnostic tests and the difficulties in conducting them in healthcare centers. In a study conducted by the French reference center for platelet disorders, a prevalence of 25% was found within a cohort of 283 patients [10]. Additionally, multiple genes related to this condition have been identified, with notable mutations in the RUNX1 and FLI1 genes. However, due to this disease's heterogeneity, multiple genes may not yet be identified [2,11,12].

The characteristic clinical presentation of these patients is a bleeding diathesis, with some cases requiring blood transfusions [2,13]. The most frequent and characteristic laboratory finding is the absence of a second wave of ADP during light transmission aggregometry. However, up to 25% of patients may not show any abnormalities in this test, as in the case of our patient [2,14]. Therefore, electron microscopy is considered the best diagnostic tool as it allows direct visualization of the decrease in the number of platelet-dense granules [15].

It is essential to characterize patients with non-syndromic dense granule deficiencies due to their association with an increased risk of bleeding. Detecting these deficiencies contributes to providing timely prophylaxis, symptomatic treatment, and follow-up [16]. In the case of our patient, the presentation was atypical as she had never experienced any signs of bleeding during childhood hemostatic challenges or previous surgical procedures. Similarly, platelet aggregation tests were normal, consistent with the literature, and confirmed that electron microscopy is the gold standard for diagnosing this condition.

Currently, the patient is stable, and although there is no cure for this condition, a timely diagnosis was made. She continues to be under follow-up and receives prophylactic management with tranexamic acid during her menstrual periods.

## Conclusions

Even though storage pool deficiencies are a relatively common cause of bleeding, especially in children, it is usually underdiagnosed due to the technical difficulties in accomplishing this. Therefore, it is essential to know the diagnosis algorithm for a child with bleeding symptoms and always bear in mind the possibility of storage pool deficiencies being the cause of the patient's clinical picture.
